# Ultrasensitive, green molecularly-imprinted poly(o-phenylenediamine) sensor on pencil graphite for trace ertugliflozin quantification in plasma and tablets

**DOI:** 10.1186/s13065-025-01681-1

**Published:** 2025-11-29

**Authors:** Menna Farrag, Sally S. El-Mosallamy, Bassam Shaaban Mohammed, Hytham M. A. Ahmed

**Affiliations:** 1 Pharmaceutical Analytical Chemistry Department, Faculty of Pharmacy, Menoufia University, Shebin Elkom, 32511 Egypt; 2 Pharmaceutical Analytical Chemistry Department, Faculty of Pharmacy, Cairo University, Kasr El Aini, Cairo, 11562 Egypt; 3 Pharmaceutical Analytical Chemistry Department, Faculty of Pharmacy, Menoufia National University, 70th Km Cairo-Alexandria Agricultural Road, Menoufia, Egypt

**Keywords:** Electrochemical sensor, Molecular imprinting, Poly(o-phenylenediamine), Electropolymerization, Ertugliflozin

## Abstract

This study presents an ultrasensitive electrochemical sensor aimed at the detection and quantification of ertugliflozin l-pyroglutamic acid (EGZ), an anti-diabetic agent, using molecularly imprinted polymer (MIP) technology. The sensor was fabricated by electropolymerizing o-phenylenediamine (o-PD) onto a pencil graphite electrode (PGE), with EGZ serving as the template molecule. The detection of EGZ was achieved through indirect sensing, where EGZ competes with the redox-active probe ferrocyanide/ferricyanide ([Fe (CN)₆]^3−/4−^) for the binding sites of the MIP. The resulting electrical signal was measured using differential pulse voltammetry (DPV). The sensor demonstrated excellent sensitivity, with a linear response range from 1 × 10⁻¹² to 1 × 10⁻¹⁰ M and a limit of detection (LOD) of 6.3 × 10⁻¹⁴ M. A non-imprinted polymer (NIP) was prepared under the same electropolymerization conditions but without the inclusion of EGZ. The NIP sensor served as a control, and the imprinting factor (IF) was determined to be 6, confirming the success of the imprinting process and the formation of selective recognition sites. Characterization of the proposed sensor was conducted using cyclic voltammetry (CV), electrochemical impedance spectroscopy (EIS), and scanning electron microscopy (SEM) coupled with energy-dispersive X-ray analysis (EDX). Additionally, the sensor successfully detected EGZ in spiked human plasma, demonstrating its practical applicability in complex biological samples. The environmental impact of the proposed method was assessed using the AGREE and GAPI green evaluation tools, which confirmed its environmentally friendly nature. Furthermore, the sensor exhibited high selectivity in the presence of commonly co-formulated drugs, such as sitagliptin and metformin, indicating its potential for pharmaceutical applications.

**Supplementary Information:**

The online version contains supplementary material available at 10.1186/s13065-025-01681-1.

## Introduction

Diabetes mellitus is a complex metabolic disorder that affects millions of people worldwide. According to the International Diabetes Federation, approximately 425 million individuals live with diabetes, 85–90% of whom suffer from type 2 diabetes (T2D). This number is expected to exceed 650 million by 2040 [1, 2]. Despite the availability of various oral and injectable medications, many T2D patients struggle to meet their glycemic targets due to poor medication adherence, which is often driven by undesirable side effects such as weight loss and hypoglycemia. Consequently, developing anti-diabetic medications that are not only effective but also well-tolerated, whether used alone or in combination with other drugs, is crucial [2, 3].

Sodium‒glucose cotransporter 2 (SGLT2) inhibitors are a promising class of insulin-independent antihyperglycemic agents that have garnered significant attention in the treatment of T2D, especially in patients who are at risk for renal or cardiovascular complications [2, 4, 5].

**Fig. 1:**
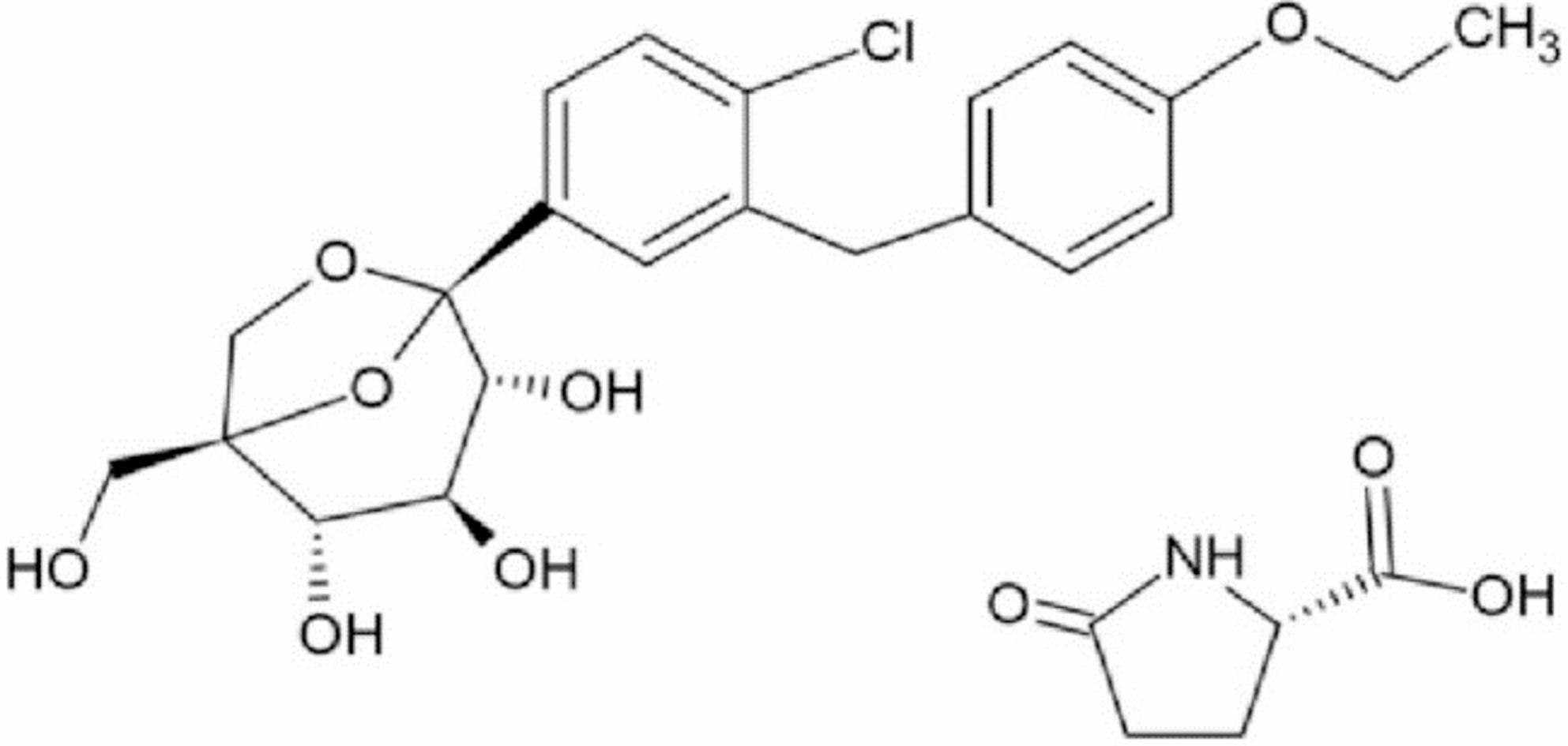
Ertugliflozin l-pyroglutamic acid chemical structure

Ertugliflozin l-pyroglutamic acid (EGZ) (Fig. 1) is the fourth drug in the SGLT2 inhibitor class and was approved by the U.S. Food and Drug Administration (FDA) in December 2017 under the brand name Steglatro^®^ for the treatment of T2D. It is available in doses of 5 mg and 15 mg [5, 6]. EGZ functions by inhibiting the SGLT2 protein, which is responsible for reabsorbing glucose in the kidneys. This action increases glucose excretion and lowers hemoglobin A1c levels by approximately 0.6% to 1.0% [7]. Therefore, developing accurate methods for detecting and quantifying EGZ in pharmaceutical formulations and biological fluids is essential.

A review of the literature reveals several analytical methods for detecting EGZ. These methods include spectrophotometry [8], spectrofluorimetry [9], RP-HPTLC [10], HPLC [11–22], UPLC [23–26], LC/MS/MS [27, 28], and GC [29]. However, to the best of our knowledge, there are no documented electrochemical methods for the determination of EGZ, despite the advantages of electrochemical techniques, which include high selectivity, sensitivity, environmental friendliness, and minimal sample preparation. These benefits have contributed to the widespread application of electroanalytical techniques across various fields, including pharmaceuticals [30, 31], biological systems [32, 33], and environmental applications [34].

This research aims to address the existing gap by using molecularly imprinted polymer (MIP) technology to develop an electrochemical sensor for the detection of EGZ. MIPs are highly selective, crosslinked polymers with three-dimensional (3D) cavities that mimic the structure of the target analyte. These polymers are created by polymerizing functional monomers in the presence of a template molecule, after which the template is removed to create specific binding sites for the analyte [35]. MIPs have gained popularity in analytical chemistry because of their stability, cost-effectiveness, ease of preparation, and reusability [36–38]. The combination of MIPs with electrochemical sensors can significantly increase selectivity, which is a crucial factor in achieving sensitive detection.

Compared with other polymerization techniques, electropolymerization offers several advantages, including the ability to conduct the process at room temperature without the need for light, oxidants, or free radicals. Furthermore, this method allows for precise control over the properties of the polymer by adjusting parameters such as current, voltage, and polymerization time [39].

o-Phenylenediamine (o-PD), an aniline derivative, is particularly well-suited for electropolymerization because of its capacity to form hydrogen bonds with the template molecule. This results in the creation of complementary cavities once the template is removed [40].

Pencil graphite electrodes (PGEs) have gained popularity in electrochemical analysis because of their low cost, user-friendly design, and disposable nature. Compared with other electrodes, PGEs offer several advantages, including an adjustable electroactive surface area, high sensitivity, reduced background currents, and good reproducibility. These features make PGEs particularly effective for detecting low concentrations of analytes without the need for sample pre-concentration or deposition steps [41].

In this study, we present the development of a voltammetric sensor for the detection of EGZ using MIP-based poly(o-phenylenediamine) (PoPD) films deposited on PGE. The performance of the sensor was evaluated through differential pulse voltammetry (DPV) by measuring the reduction in the ferrocyanide/ferricyanide ([Fe (CN)_6_]^3−/4−^) redox probe signal upon EGZ binding. The sensor was further characterized by scanning electron microscopy (SEM), energy-dispersive X-ray analysis (EDX), cyclic voltammetry (CV), and electrochemical impedance spectroscopy (EIS). To assess the environmental impact of the proposed method, we employed the analytical greenness metric (AGREE) and the green analytical procedure index (GAPI) to evaluate its greenness both qualitatively and quantitatively [42, 43].

## Experimental

### Instruments

All the electrochemical measurements were conducted using an Autolab potentiostat/galvanostat (model: PGSTAT204, Metrohm, Netherlands), which was controlled by NOVA version 1.11 software. Data processing and recording were performed through a computer interface. The electrochemical setup included a three-electrode system: an Ag/AgCl reference electrode, a platinum wire counter electrode, and a pencil graphite electrode (PGE, 2B, 0.7 mm, DONG-A, South Korea) as the working electrode. pH measurements were monitored using a Jenway pH glass electrode (model: P14/BNC, Bibby Scientific Ltd., UK). Spectrophotometric measurements were performed using a UV‒visible double-beam spectrophotometer (model: 1610, Shimadzu, Japan).

### Chemicals and reagents

Ertugliflozin l-pyroglutamic acid (EGZ), with a molecular weight of 566 g/mol and a purity of 100.6% (according to the reported method [9]), was graciously provided as a gift by Hikma Pharma S.A.E. located in the 6th of October city, Egypt. It served as the standard for the study, and all concentrations were expressed based on the salt form. o-Phenylenediamine (o-PD), potassium ferrocyanide (K_4_[Fe (CN)_6_]), potassium ferricyanide (K_3_[Fe (CN)_6_]), and ethanol were purchased from Sigma-Aldrich (Darmstadt, Germany). Potassium chloride, acetic acid and sodium hydroxide were acquired from LobaChemie (India). All the chemicals and solvents used in this work were of analytical grade and did not require further purification. Double distilled water was generated using an automatic water still (Sci Finetech, Seoul, South Korea). A 0.1 M phosphate buffer solution (PBS) was prepared using dipotassium hydrogen phosphate and sodium chloride maintaining a pH range of 6‒8.5 [44]. Blank human plasma was supplied by Vaccines and Sera Company (VACSERA, Cairo, Egypt).

### Pharmaceutical dosage forms

Glibafloz tablets^®^ (B.N:001), each containing 15 mg of EGZ, were manufactured by Hikma Pharma S.A.E. (6th of October City, Egypt), and obtained from a local pharmacy.

### Preparation of MI-PoPD modified PGE

Cyclic voltammetry was employed to electropolymerize o-PD onto the PGE surface. Initially, the surface of the bare PGE was cleaned with a water–methanol mixture, followed by drying with a stream of nitrogen. Next, the pretreated PGE was immersed in a phosphate buffer solution (pH 7) containing 10 mM of both EGZ and o-PD. The solution was purged with nitrogen gas for approximately 15 min to eliminate any dissolved oxygen. Subsequently, ten voltammetric cycles were conducted within a potential range of − 0.1 V to 1 V at a scan rate of 25 mV/s to carry out the electropolymerization process. After that, the electropolymerized electrode was cleaned with water and soaked in a washing solution composed of ethanol and acetic acid (8:2, v/v, total volume 10 mL) for 20 min under mild shaking to extract the template (EGZ) from the MIP film. Following the washing step, the MI-PoPD/PGE sensor was immersed in a redox probe solution containing 10 mM [Fe (CN)_6_]^3−/4−^ in 0.1 M KCl, and differential pulse voltammetry (DPV) measurements were recorded. The measurements that correspond to the peak current height of [Fe (CN)_6_]^3−/4−^ after washing are referred to as the after-wash response (*I*^*o*^).

Finally, for the rebinding of EGZ and subsequent measurements, the sensor was incubated with the target EGZ sample for 20 min. The measurements taken after this step, corresponding to the peak current height of [Fe (CN)₆]^3−/4−^ following EGZ rebinding, are referred to as (*I)*.

Additionally, a non-imprinted polymer (NIP) electrode was prepared under the same conditions but without the inclusion of EGZ.

### Procedures

#### Electroanalytical measurement of EGZ

The optimized parameters for DPV were set as follows: step potential, 5 mV; scan rate, 10 mV/s; interval time, 0.5 s; modulation time, 0.05 s; and a modulation amplitude, 25 mV. All the measurements were conducted at room temperature. Prior to analysis, the MI-PoPD/PGE sensor was cleaned with distilled water after being incubated in the specified concentration of EGZ for 20 min while gently shaking. Using DPV at room temperature, within a potential range of -0.4 V to 1 V, EGZ was indirectly quantified by recording the decrease in the redox-active probe ([Fe (CN)_6_]^3−/4−^) (*I*), which resulted from the occupation of the imprinted cavities by EGZ molecules. To prepare a concentration range of EGZ (from 1 × 10^−12^ − 1 × 10^−10^ M), different aliquots of the stock solution were diluted with phosphate buffer at pH 6. The current was calculated for each EGZ concentration on the basis of DPV records. A calibration curve was then constructed to illustrate the correlation between the EGZ concentrations and the ratio of the difference in peak current height, expressed as *(I⁰–I)/I⁰*, for the [Fe (CN)_6_]^3−/4−^ redox probe.

#### Application to dosage form

Five Glibafloz Tablets^®^, each containing 15 mg of EGZ, were finely ground into a powder and thoroughly mixed. An amount equivalent to 22.4 mg of EGZ was accurately weighed and transferred to a 25 mL volumetric flask, after which 12.5 mL of ethanol was added to dissolve the powder. After that, the flask was sonicated for 45 min and diluted to the mark with ethanol, after which the contents were mixed thoroughly. The solution was then filtered through filter paper. Serial dilutions were prepared with a phosphate buffer solution (pH 6) to obtain the desired concentrations in the range of 1 × 10^−12^ to 1 × 10^−10^ M. The procedure for “*electroanalytical measurement of EGZ” was followed* Differential pulse voltammograms were recorded using the previously optimized parameters, and the EGZ concentration was determined.

#### Biological matrix application (spiked human plasma)

One milliliter of blank human plasma was mixed with three milliliters of acetonitrile in a set of six centrifuge tubes. Each tube was spiked with different aliquots of EGZ. The mixture was sonicated for 15 min and then centrifuged at 4000 rpm for 45 min. After centrifugation, the supernatant was collected and evaporated at 60 °C. The residue was then reconstituted with 15 mL of phosphate buffer (pH 6), resulting in final concentrations of 2 × 10⁻¹², 4 × 10⁻¹², 6 × 10⁻¹², 1 × 10⁻¹¹, 4 × 10⁻¹¹, and 8 × 10⁻¹¹ M. Each concentration was measured in triplicate following the procedure outlined above.

## Results and discussion

### UV-Spectrophotometric technique for studying EGZ (template) and o-PD (monomer) interaction

To investigate the optimal interaction between the template (EGZ) and the monomer (o-PD), a double-beam UV spectrophotometer was utilized, and different pH values were tested. As illustrated in Fig. S1. (available in the supplementary information), the spectra of 1 × 10^–4^ M of EGZ and o-PD, each measured individually, as well as a mixture of their equimolar solutions in phosphate buffer solution (pH 6, pH 7 and pH 8.5), and the calculated mixture were analyzed. Notably, the spectrum of the EGZ/o-PD mixture displayed a pronounced hyperchromic effect in phosphate buffer at pH 7, indicating the formation of a stable complex with o-PD [45, 46]. Consequently, phosphate buffer at pH 7 was chosen for the electropolymerization process in this study.

### o-Phenylenediamine electropolymerization on the surface of PGE

**Fig. 2:**
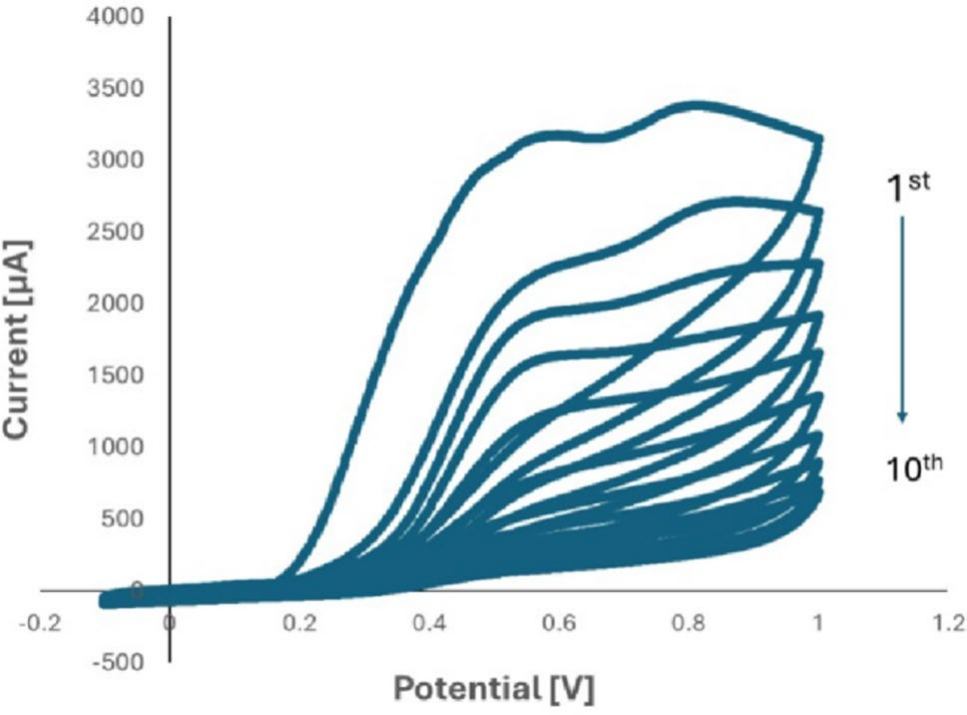
o-phenylenediamine electropolymerization and ertugliflozin l-pyroglutamic acid using cyclic voltammograms on pencil graphite electrodes with optimal parameters of 10 cycles, phosphate buffer pH 7, scan rate (25 mV/s)

To the best of our knowledge, the literature review indicates that there have been no previous electrochemical studies on the determination of EGZ; in particular, no studies have demonstrated the electropolymerization of o-PD on the surface of PGE for its detection. Figure 2 presents the cyclic voltammogram of 10 cycles, using a potential range from − 0.1 to 1 V at a scan rate of 25 mV/s, illustrating the electropolymerization of o-PD on the PGE surface in the presence of the EGZ. Upon application of this voltage, o-PD molecules undergo intermolecular cyclization, oxidation, and polymerization, resulting in a structure resembling phenazine [45]. This is demonstrated by the rapid decrease in the anodic peak current after the first cycle, indicating a high rate of electropolymerization. As the number of cycles increases, the anodic current peak decreases steadily until it disappears after 10 cycles, indicating the formation of an adhesive PoPD polymeric coating that completely insulates the PGE surface [47–49]. PoPD/PGEs were fabricated through electropolymerization using EGZ as a template molecule. A non-imprinted polymer (NIP) was prepared under the same electropolymerization conditions as the MIP but without the presence of EGZ, serving as a control.

### Optimization conditions for the MI-PoPD sensor preparation

To achieve the highest sensitivity, various parameters of the proposed method were fine-tuned through systematic optimization. In each experiment, one parameter was varied while all other conditions remained constant.

#### Optimization of electropolymerization and rebinding pH

**Fig. 3:**
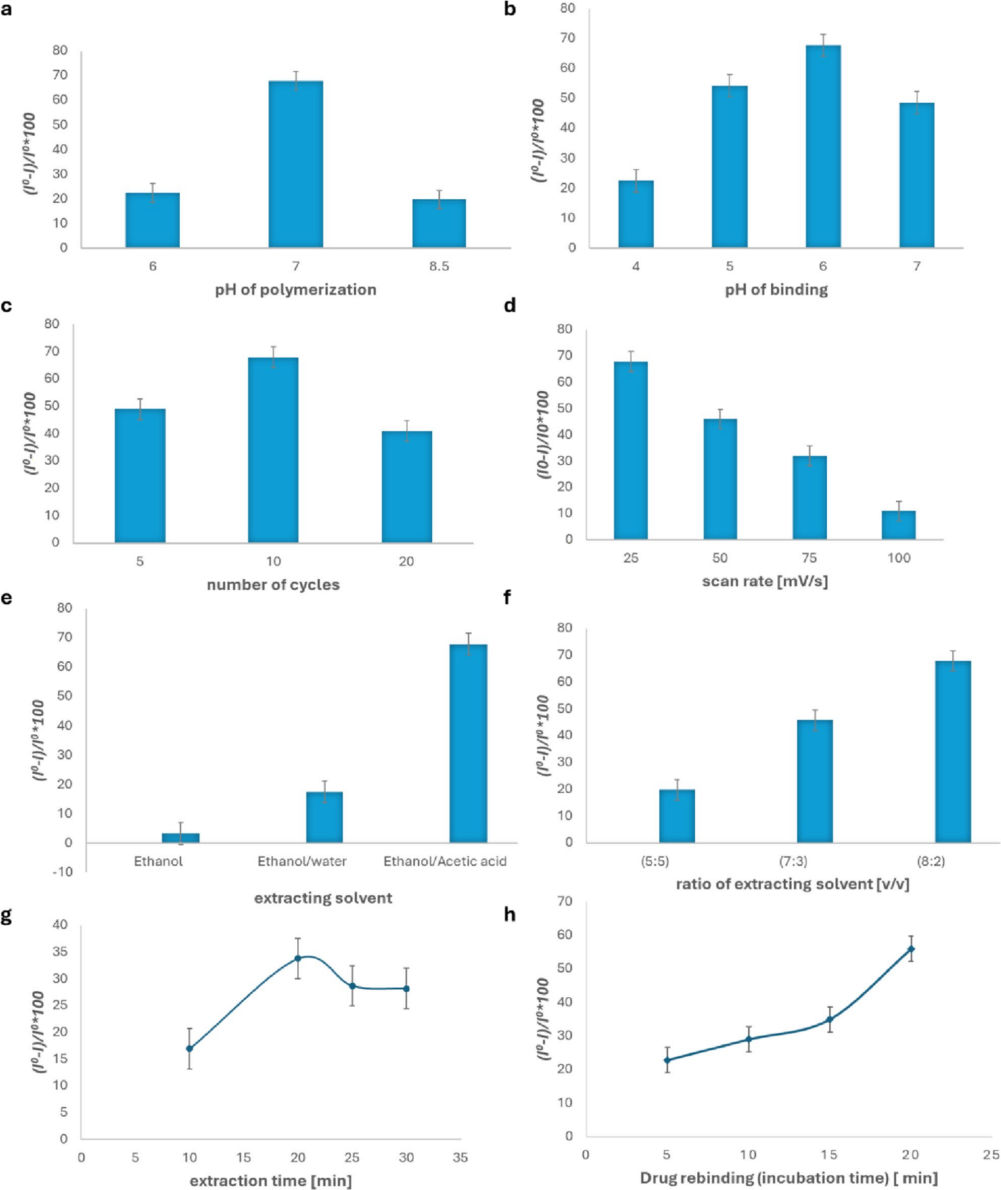
Optimization of various parameters influencing the electropolymerization of o-phenylenediamine using differential pulse voltammetry (DPV) with the following conditions: step potential = 5 mV, interval time = 0.5 s, modulation time = 0.05 s, and modulation amplitude = 25 mV. The effects of **a** polymerization pH, **b** binding pH, **c** number of polymerization cycles, **d** scan rate of electropolymerization, **e** type of extraction solvent, **f** extraction solvent ratio, **g** extraction time, and **h** incubation time were investigated. Each measurement represents the mean of three replicates (*n* = 3). In all experiments, only one parameter was varied at a time, while the others were kept constant

The electropolymerization of o-PD and its interaction with the EGZ template are influenced by the pH of the phosphate buffer solution [47]. To optimize the electropolymerization process and the interaction between o-PD and EGZ, different pH values ranging from 4 to 8.5 were prepared. This also allowed for an investigation into how pH affects the electrochemical response of the MIP sensors. As shown in Fig. 3a, a pH value of 7 resulted in the greatest percentage reduction in the current peak height. Consequently, pH 7 was selected as the optimal condition for the electropolymerization process.

After washing, the template molecules are removed, exposing the binding cavities, which are then ready to rebind to the target molecule (EGZ). This rebinding step is significantly influenced by pH. Therefore, various pH values were tested, and as shown in Fig. 3b, the greatest percentage reduction in the current peak height occurred at pH 6. Consequently, pH 6 was selected as the optimal pH for the rebinding process.

#### Optimization of electropolymerization: number of cycles

The number of cycles is a crucial factor influencing the electropolymerization process, as the thickness of the MIP film significantly affects the current response of the MIP sensor. This film thickness can be controlled by adjusting the number of cycles during electropolymerization [45, 47, 50]. The effect of number of cycles was investigated by calculating the percentage decline in the current peak height using the formula ((*I⁰−I /I⁰*) * 100) at various numbers of cycles (5, 10, and 20 cycles). As shown in Fig. 3c, the optimum number of cycles was determined to be 10, as it resulted in the greatest reduction in the current peak height. Therefore, 10 cycles were selected for the electropolymerization process.

#### Optimization of electropolymerization: scan rate

Various scan rates were tested to optimize the electropolymerization process. As shown in Fig. 3d, the greatest reduction in the current peak height% occurred at 25 mV/s; therefore, a scan rate of 25 mV/s was selected as the optimum value for electropolymerization.

#### Optimization of extraction solvent

There are several methods for extracting template molecule from MIPs. These methods include overoxidation of the polymer [51], supercritical fluid desorption [52], and redox of the template within the polymer [53]. However, the most commonly used method is solvent extraction [54], which is used in this study. In this method, the interaction between solvents (either one solvent or a mixture of two solvents) and the polymer is strong, facilitating the removal of the template molecule [55]. Various extraction solvents, such as ethanol, a mixture of ethanol and water, and ethanol mixed with acetic acid, were studied. Among these mixtures, the ethanol: acetic acid mixture demonstrated the highest efficacy for template removal, as shown in Fig. 3e. Therefore, ethanol: acetic acid was chosen as the optimal extraction solvent.

#### Optimization of ratio of extraction solvent

Various ratios of ethanol to acetic acid (5:5, 7:3, and 8:2, v/v) were tested as extraction solvents. As shown in Fig. 3f, the optimal ratio of ethanol to acetic acid was 8:2 (v/v), which was therefore selected for subsequent experiments.

#### Optimization of extraction time

Extraction time is a crucial factor in the template extraction process. It was optimized by immersing the electrodes in an 8:2 (v/v) solution of ethanol and acetic acid for 10, 20, 25, and 30 min, respectively. As revealed in Fig. 3g, effective template removal was achieved after an extraction time of 20 min.

#### Optimization of incubation time

One of the main factors influencing the rebinding ability of the template molecule is the incubation period of the MIP sensor in the target molecule solution [56]. To examine how the incubation time affects the response of the MI-PoPD sensor, the sensor was incubated for various durations, ranging from 10 to 20 min, in a solution containing 1 × 10^−5^ M of the target molecule, EGZ. The effect was assessed by calculating the percentage reduction in the current peak height at each time. As shown in Fig. 3h, an extraction time of 20 min demonstrated the highest adsorption of EGZ.

### Electroanalytical measurement of EGZ using the suggested MI-PoPD

**Fig. 4:**
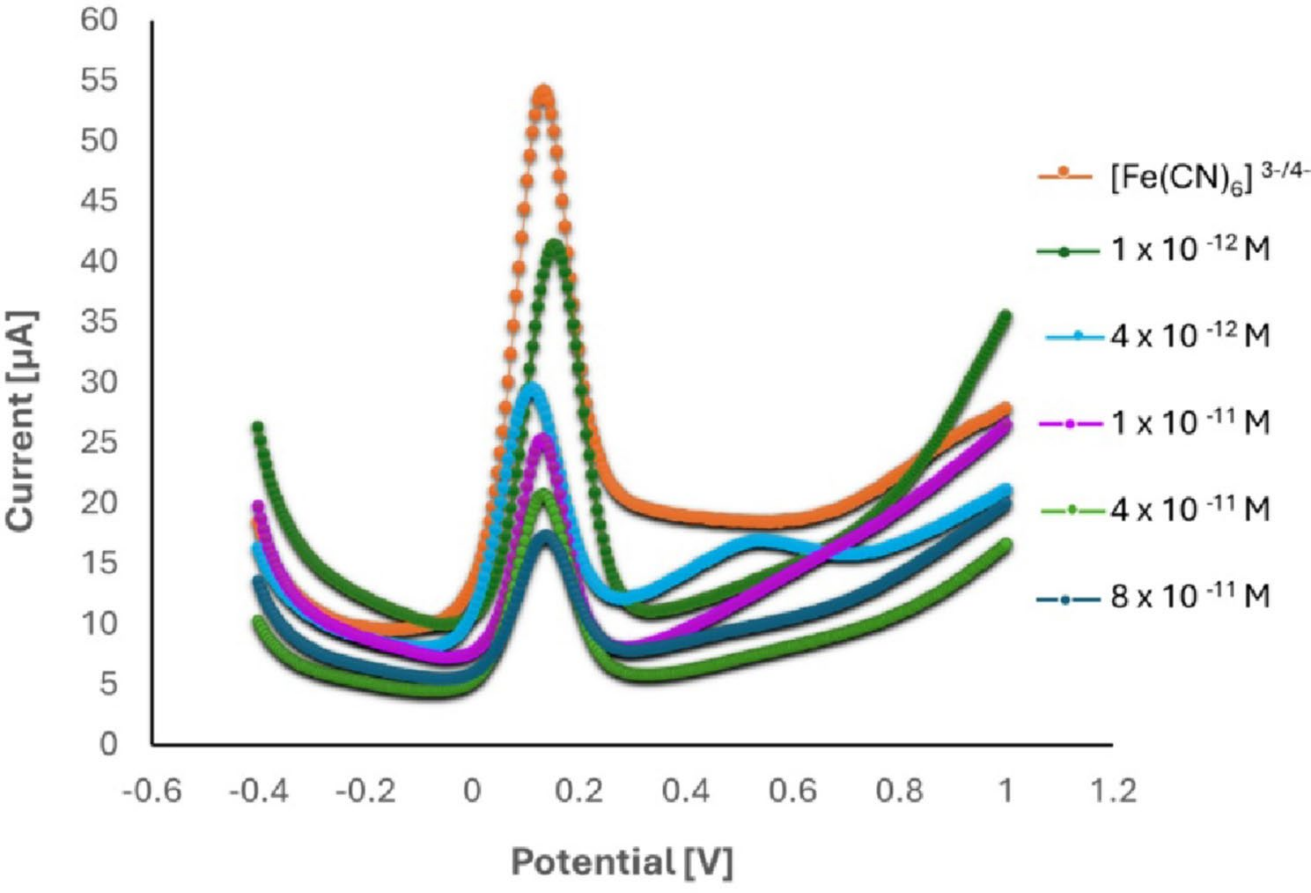
Differential pulse voltammograms of poly(o-phenylenediamine)/pencil graphite electrode in 10 mM [Fe (CN)_6_]^3−/4−^ redox probe following washing and incubation in varying ertugliflozin concentrations (1 × 10^−12^ – 8 × 10^−11^ M), with a scan rate of 10 mV/s, over a potential range from − 0.4 to 1.0 V

In this study, EGZ was quantified indirectly using the proposed MI-PoPD. [Fe (CN)6]^3−/4−^ was utilized as an electroactive redox probe to measure the uptake and release of EGZ into and from the imprinted recognition sites. When all the recognition sites are occupied by EGZ, the redox probe cannot access them, resulting in a low voltammetric signal. However, when the recognition sites are open (i.e., not bound to EGZ), the redox probe generates its normal voltammetric signal. Furthermore, the uptake and release of [Fe (CN)_6_]^3−/4−^ can be continuously monitored electrochemically by observing the competition between the electro-active probe [Fe (CN)_6_]^3−/4−^ and the electro-inactive EGZ (Fig. 4). The binding properties of the MIP-modified electrode were studied using DPV data acquired from the calibration range between $\:{10}^{-10}$ and $\:{10}^{-12}$  M. The change in the current response of the [Fe (CN)_6_]^3−/4−^ redox probe $\:({I}_{0}-I)$ was calculated by subtracting the current recorded in the presence of EGZ $\:\left(I\right)$ from that recorded in its absence $\:\left({I}_{0}\right)$. The $\:{K}_{D}$ value was obtained from the following model equation:

$${I_0} - i=\frac{{{B_{MAX}}\,C}}{{{K_D}+c}}+{N_S}\,c$$

where $\:C\:$ is the bulk concentration of the target, $\:{B}_{\text{max}}\:$ is the maximum number of binding sites in the MIP, $\:{K}_{D\:}$ is the equilibrium dissociation constant, and $\:{N}_{S\:}$ is the binding constant for nonspecific adsorption. The value K_D_ obtained from the fitting was 4.8231 × 10^−12^ M (R2 = 0.989), confirming that EGZ had a high affinity for the recognition sites in the MIP.

The sensor based on the NIP showed a much lower current response compared to the MIP, and the calculated imprinting factor (IF) was 6, confirming the successful imprinting process and the formation of selective recognition sites.

### Surface characterization of the MI-PoPD film

#### Electrochemical characterization of the imprinted sensor

Cyclic voltammetry (CV) and electrochemical impedance spectroscopy (EIS) were employed to evaluate the performance of the imprinted sensor. As documented in the literature, CV is one of the most fundamental techniques for the electrochemical characterization of electrodes [57–59].

**Fig. 5:**
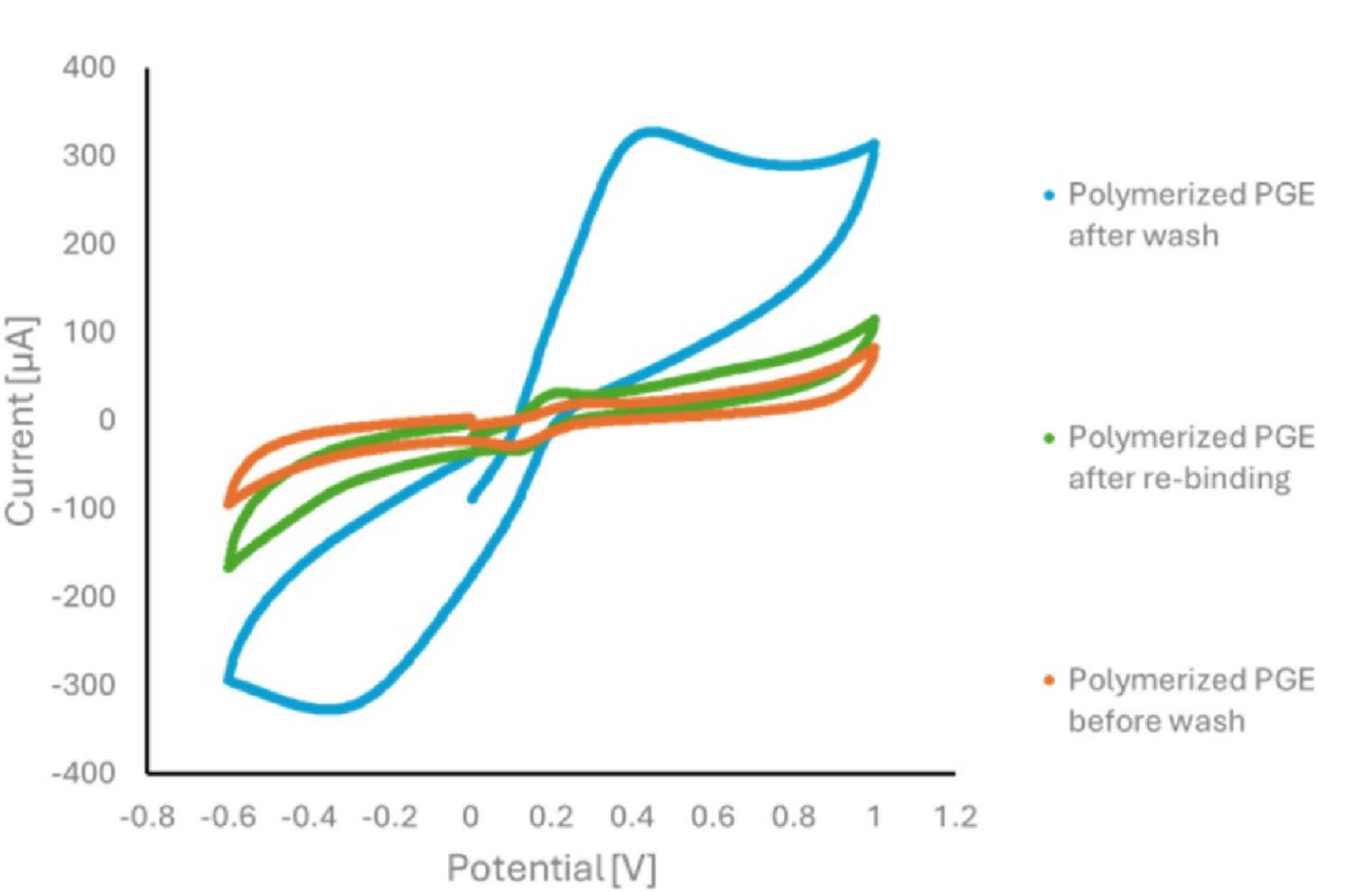
Cyclic voltammograms of the proposed sensor with a redox probe solution of 10 mM [Fe (CN) _6_]^3−/4−^ in 0.1 M KCL, at a scan rate of 0.1 V/s, over a potential range from − 0.6 to 1.0 V

The cyclic voltammetry process is shown in Fig. 5. The peak current value of the proposed MI-PoPD sensor before template removal was significantly reduced as a result of electropolymerization, indicating complete coverage of the PGE surface with a polymeric film, which blocks electron transfer. However, after the MI-PoPD was washed with the extracting solvent (ethanol: acetic acid (8:2 v/v)), the peaks of the [Fe (CN)_6_]^3−/4−^ redox probe reappeared. This observation can be attributed to the removal of the template (EGZ), which generated cavities in the PoPD matrix, allowing the probe to pass through them as active channels. The response of [Fe (CN)_6_]^3−/4−^ then noticeably decreased again because the channels were blocked once more after rebinding with EGZ. The electroactive surface area of the electrode after each step was calculated as follows: bare PGE electroactive surface area = 0.188 cm^2^, polymerized PGE electroactive surface area = 0.008 cm^2^, washed PGE electroactive surface area = 0.141 cm^2^ and after rebinding PGE electroactive surface area = 0.013 cm^2^.

**Fig. 6:**
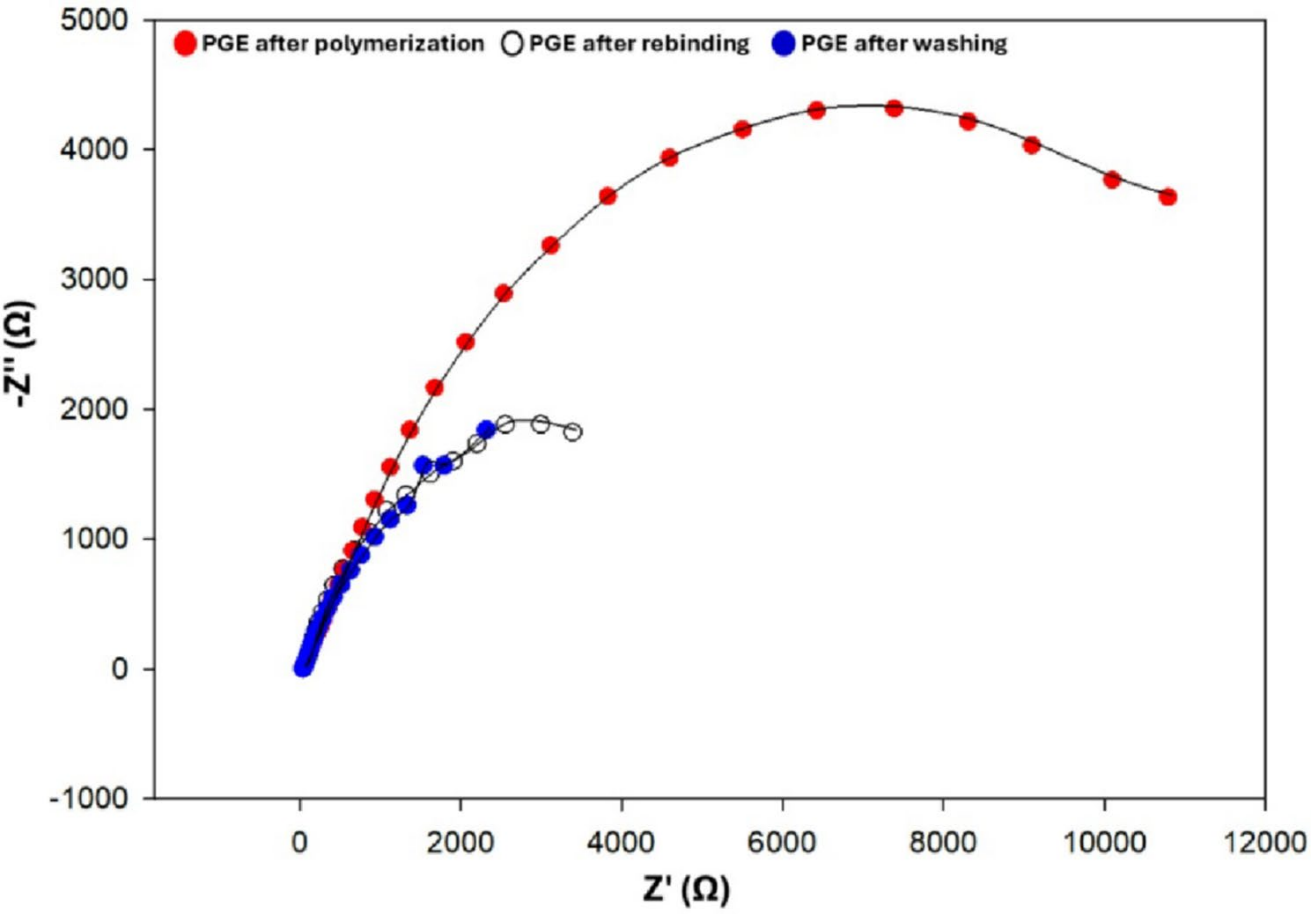
Nyquist plots of the proposed electrode after polymerization, washing, and rebinding, respectively, recorded in 10 mM [Fe (CN)_6_]^3−/4–^ containing 0.1 M KCl over a frequency range of 100,000 kHz to 100 mHz with an applied AC signal of 5 mV (rms). The dots represent the experimental (raw) data, while the solid lines correspond to the fitted curves obtained using the Randles equivalent circuit

To further investigate the electrode surface coating, the EIS technique was employed, with a 10 mM solution of [Fe (CN)_6_]^3−/4–^ in 0.1 M KCl used as the redox probe at a frequency range of 100 kHz to 100 mHz, with an AC amplitude of 10 mV (rms) and approximately 9 points per decade. The validity of the data was confirmed via Kramers–Kronig (KK) transformation. To estimate the circuit components, the results were fitted to Randles’ equivalent circuits utilizing the Nova 1.11.0 software. Owing to the insulating characteristics at the interface between the electrode and electrolyte, the semicircle diameter was equivalent to the charge transfer resistance (R_ct_). As shown in Fig. 6, the R_ct_ of the imprinted sensor was initially high (11350.05 Ω) immediately after polymerization and before template elution, indicating the presence of a thick polymer layer. The R_ct_ drastically decreased to 2919.59 Ω after template removal, reflecting the formation of binding cavities. Following incubation in the EGZ solution, the R_ct_ increased again to 3804.79 Ω, confirming the successful rebinding of EGZ to the imprinted cavities.

#### Scanning electron microscopy (SEM) combined with energy-dispersive X-ray analysis (EDX)

The surface morphologies of the prepared PGEs were analyzed using SEM and EDX to obtain crucial information. The surface of bare PGE (BPGE) displayed thin and delicate graphite layers, characterized by a flat and smooth appearance (Fig. S2a). In contrast, the surface morphology of the PGE surface was significantly altered following the electrochemical polymerization of o-PD. This process resulted in the development of a rough, porous, and grainy polymeric film on the PGE surface (Fig. S2b). This distinct morphological structure provides clear evidence that o-PD monomeric units were successfully electropolymerized on the PGE surface. SEM measurements revealed that the thickness of the electropolymerized film was approximately 15.36 μm.

Additionally, EDX analysis was conducted on both electrodes to provide further evidence for the observed morphological changes. The elemental composition of BPGE included carbon (C, 83.26%), oxygen (O, 12.77%), silicon (Si, 0.49%), and calcium (Ca, 1.81%), as shown in Fig. S2c. These elemental signals indicate the presence of clay and graphite crystals in the pristine PGE structure. Furthermore, an additional signal for nitrogen (N, 11.55%) was observed exclusively in the PoPD/PGE electrode (Fig. S2d). This nitrogen signal is attributed to the amine (–NH_2_) functional group of o-PD, providing clear evidence that poly(o-PD) films were successfully formed on the PGE surface.

### Method validation

Under optimal electrochemical conditions, the method demonstrated a linear response with a correlation coefficient (r) of 0.9999 over the range of 1 × 10^−12^ to 1 × 10^−10^ M (Fig. S3.). A variety of validation parameters were calculated following the ICH guidelines [60], and the results are summarized in Table 1. Positive outcomes were observed (*n* = 9) when assessing the intraday and interday precision, with % RSD values below 2%, demonstrating the precision of the proposed method.

**Table 1:** Validation parameters of the proposed differential pulse voltammetric method for determination of ertugliflozin l-pyroglutamic acid

Parameters	EGZ
Concentration range (M)	1 × 10^−12^ – 1 × 10^−10^
Correlation coefficient (r)	0.9999
Slope ± SD*	8 × 10 ^9^ ± 0.00015
Intercept	0.1621
Accuracy^a^ (mean ± SD)	99.9 ± 1.4
Intraday precision^b^ (% RSD)	1.01
Interday precision^b^ (% RSD)	1.58
LOD (M)^c^	6.3 × 10^−14^
LOQ (M)^d^	1.91 × 10^−13^

^*^Average of three determinations

^a^ Mean of five determinations

^b^ Relative standard deviations for three concentrations (1 × 10^−12^ & 4 × 10^−12^ and 1 × 10^−11^ M) were measured in triplicate on the same day for intra-day precision and on three different days for inter-day precision

^c^ LOD = 3.3 × SD of intercept/slope

^d^ LOQ = 10 × SD of intercept/slope

#### Selectivity of the fabricated MI-PoPD sensor

**Fig. 7:**
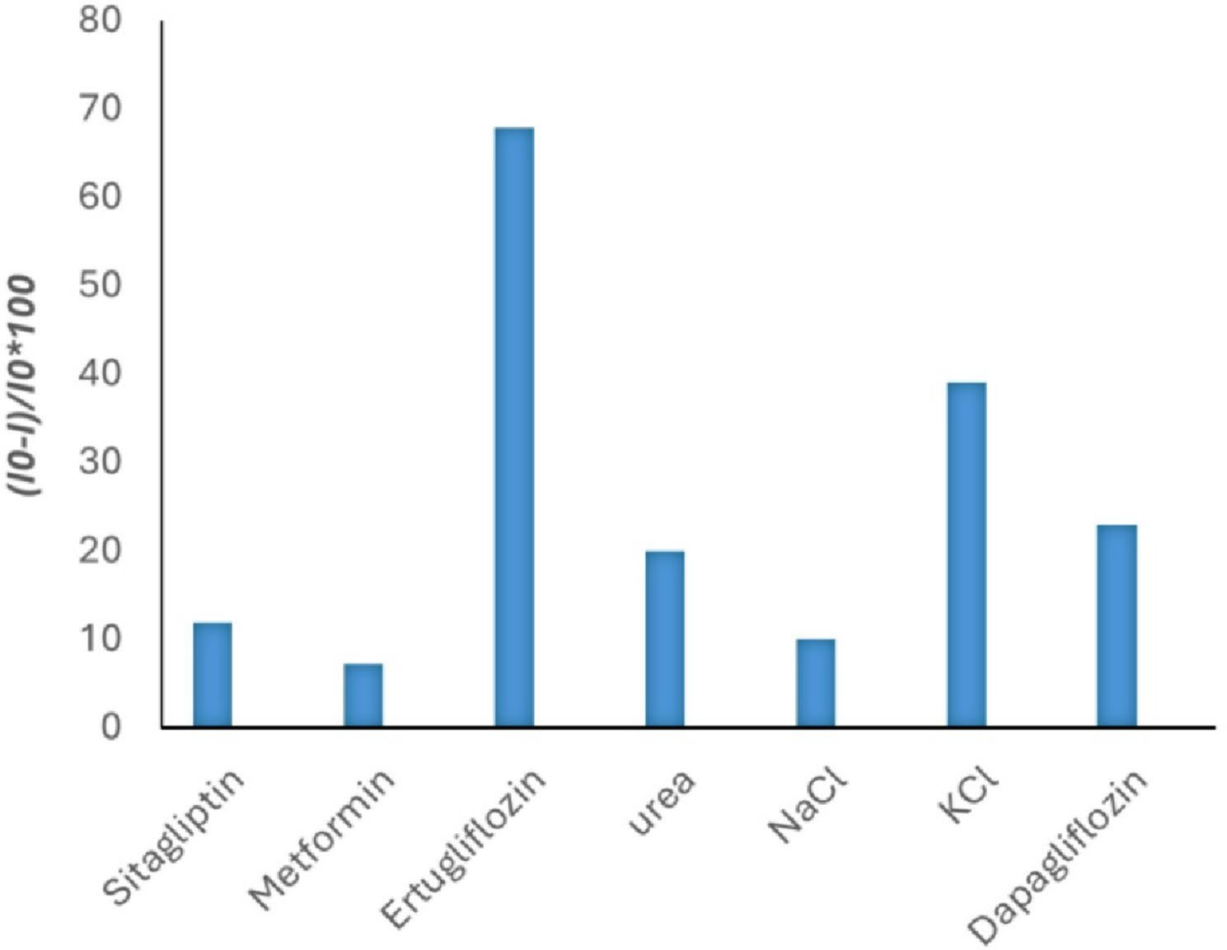
Selectivity evaluation of the fabricated sensor for ertugliflozin toward interfering analytes

To evaluate the selectivity of the fabricated MI-PoPD sensor, co-administered drugs—specifically, sitagliptin and metformin—were selected as potential interfering agents. In addition, various other interfering analytes, including dapagliflozin, a structurally analogous anti-diabetic drug, as well as other possible interfering substances commonly found in biological samples or tablet excipients, have been investigated. The MI-PoPD sensor was tested using DPV after being immersed in solutions containing EGZ and various interfering analytes, each of which was prepared at the same concentration of 1 × 10^−5^ M under identical experimental conditions. The percentage reduction in the current peak height was then measured. Furthermore, the sensor performance was evaluated in laboratory-prepared binary mixtures of EGZ with metformin, dapagliflozin, and sitagliptin. As shown in Fig. 7 and summarized in Table 2, the response signal for EGZ obtained using the MI-PoPD sensor was significantly greater than that of the interfering analytes, confirming its high selectivity. Additionally, as shown in Table 3, the fabricated sensor demonstrated excellent selectivity and practical applicability, as evidenced by recovery values of approximately 100.03%, 102.09%, and 102.94% for EGZ when mixed with sitagliptin, metformin, and dapagliflozin, respectively. These findings indicate that the MI-PoPD sensor has high selectivity toward EGZ, likely due to the structural complementarity between the imprinting sites in the MI-PoPD film and the template EGZ molecule.

**Table 2:** Selectivity evaluation of the fabricated sensor for ertugliflozin l-pyroglutamic acid toward interfering analytes using differential pulse voltammetric technique

Analyte	Concentration (M)	Current response (Io−I/Io) *100 using MI-PoPD/PGE (%)
Sitagliptin	1 × 10^–5^	11.9
Metformin		7.14
Ertugliflozin		67.88
Urea		20
NaCl		10
KCl		39
Dapagliflozin		23

**Table 3:** Selectivity evaluation of the fabricated sensor in a laboratory-prepared mixture containing ertugliflozin, l-pyroglutamic acid, and potential interfering agents using the differential pulse voltammetric technique

Lab mixture	Ratio	Concentration (M)	% recovery
Ertugliflozin and sitagliptin	1:1	1 × 10^−11^	100.03
Ertugliflozin and metformin	1:1	1 × 10^−11^	102.09
Ertugliflozin and dapagliflozin	1:1	1 × 10^−11^	102.94

#### Precision of the MI-PoPD sensor

The precision of the sensor was evaluated by measuring EGZ solutions at concentrations of 4 × 10⁻¹¹ M and 1 × 10⁻¹¹ M. Five different electrodes were used under identical conditions for these measurements. The resulting relative standard deviation (RSD) was 2.56%, indicating that the MIP sensor exhibited good precision.

#### Reusability of the proposed MI-PoPD sensor

The proposed MIP-based electrochemical sensor is designed for multiple uses rather than being a single-use sensor. After the initial binding of EGZ, the sensor maintained ≥ 90% of its original signal response for up to four consecutive adsorption–elution cycles, demonstrating excellent regeneration capability and reusability.

### Analysis of EGZ in spiked human plasma samples

The high sensitivity of the proposed MI-PoPD/PGE sensor makes it suitable for the analysis of EGZ in spiked human plasma. It has been reported that after 15 mg of ertugliflozin is taken orally once daily, the mean peak plasma concentration (C_max_) of EGZ is 268 ng/mL [6]. The proposed sensor had a very low limit of detection (6.3 × 10^−14^ M), which was significantly less than C _max_. Table 4 demonstrates the sensor’s successful application in determining EGZ in spiked plasma samples without any interference.

**Table 4:** Application of the proposed differential pulse voltammetric method using the proposed sensor for estimation of ertugliflozin l-pyrogluztamic acid in spiked human plasma

Spiked human plasma	
Conc (M)	Recovery%
2 × 10^−12^	101.25
4 × 10^−12^	99.38
6 × 10^−12^	98.54
1 × 10^−11^	101.63
4 × 10^−11^	101.03
8 × 10^−11^	100.52
Accuracy (mean ± SD)	100.39 ± 1.19

### Analysis of EGZ in pharmaceutical dosage forms

Ertugliflozin was quantified using the developed MI-PoPD/PGEs in its commercially available dosage form, Glibafloz^®^ tablets. As shown in Table 5, measurements of three distinct samples yielded an acceptable average recovery percentage with a standard deviation of less than 2. These findings demonstrate the sensor’s accuracy and reliability in measuring EGZ in the drug formulation.

**Table 5:** Application of the proposed differential pulse voltammetric method, and standard addition technique using the proposed sensor for estimation of ertugliflozin l-pyroglutamic acid in pharmaceutical dosage form

Pharmaceutical preparation	Found%^*^ ± SD	Standard addition			
		Added concentration (M)	Found concentration (M)		Recovery %
Glibafloz ^®^ tablet (Batch Number: 001)	101.13 ± 1.25	2 × 10^−12^	1.99 × 10^−12^		99.5
		4 × 10^−12^	4.05 × 10^−12^		101.25
		5 × 10^−12^	4.99 × 10^−12^		99.8
		Mean ± SD			100.18 ± 0.94
		%RSD			0.93

*Average of three determinations

Additionally, the standard addition method was applied, and the results revealed appropriate recovery percentages, as presented in Table 5. These findings emphasize the enhanced affinity of the proposed MIP sensor for EGZ recognition. The standard addition method was used to further validate the sensor’s accuracy and dependability in quantifying EGZ within complex matrices such as pharmaceutical formulations.

A comparison was made between the MIP-based electrochemical method and other reported methods to further evaluate the proposed method. Table S4. demonstrates a comparison between the proposed method with other spectrofluorimetric and chromatographic methods.

### Greenness evaluation of the proposed method

In 2000, green analytical chemistry (GAC) was introduced with the aim of reducing or completely eliminating the negative impacts of the analytical processes on both the environment and operators. However, achieving an appropriate balance between analytical performance and the desired level of greenness remains a challenge [43]. The analytical-eco Scale [61] and the national environment method index labeling (NEMI) are two tools that have been reported for assessing the greenness of analytical methods. These tools offer the advantages of presenting results in an easy-to-interpret pictogram, but they also have limitations. For instance, the NEMI provides only a qualitative evaluation of the tested method, whereas the analytical eco-scale does not explain the underlying causes of the environmental impacts associated with the analytical process [43]. Therefore, two additional tools were employed to evaluate the greenness of the proposed electrochemical method: the Analytical GREEnness metric (AGREE) [42]and the Green Analytical Procedure Index (GAPI) [43]. Together, these tools allow for both qualitative and quantitative assessments of the method’s greenness.

#### Green analytical procedure index (GAPI)

**Fig. 8:**
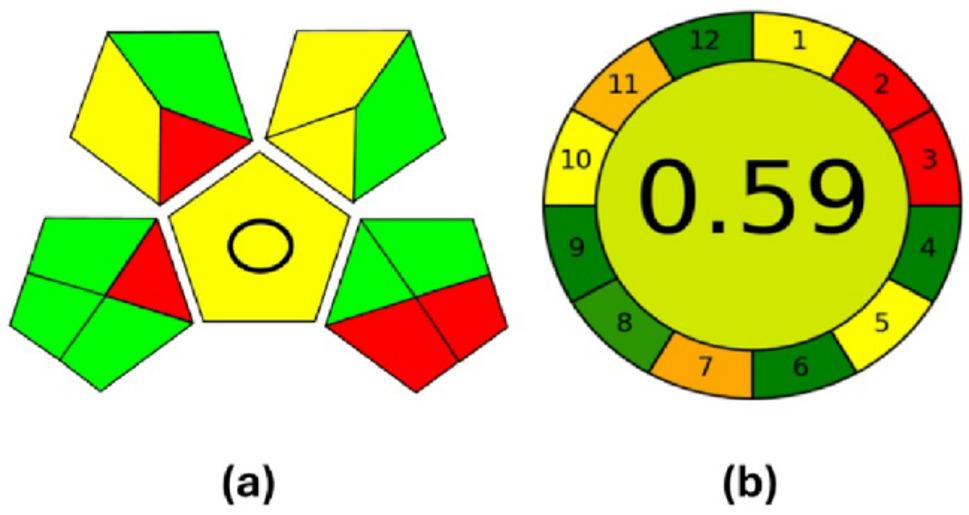
Green Analytical Procedure Index (**a**) and Analytical GREEnness metric (**b**) assessment of the proposed method’s greenness

The GAPI tool assesses the environmental impact of each stage of analytical process by using a color scale with three levels of assessment. At each step, GAPI evaluates the method’s environmental impact and greenness using a specific pictogram, which is represented by a five-pointed star symbol. The colors red, yellow, and green indicate high, medium, and low environmental impact, respectively. Wasylka provided an explanation of the GAPI parameters [43]. Figure 8a shows the GAPI assessment of the proposed method’s greenness, with additional details provided in Table S5.

#### Analytical greenness metric (AGREE)

In June 2020, the AGREE tool was introduced [42]. This tool uses downloadable software to calculate a greenness score for different methods. The final score is displayed in the center of a diagram and is based on the twelve fundamental principles of GAC. The AGREE score ranges from 0 to 1, with a score closer to one indicating a greener method, whereas a score closer to 0 signifies insufficient greenness. The software automatically generates a twelve-segment pictogram that shifts in color from green to red represent the results visually. Figure 8b shows that the suggested method achieved a score of 0.59.

## Statistical analysis

A statistical comparison between the results of the proposed method and those of the reported method [9] was performed, and the data presented in Table S6. shows that the calculated t and F values are lower than the corresponding tabulated values. This finding indicates that there is no statistically significant difference in terms of accuracy and precision between the two methods.

## Conclusion

In this study, we developed an ultrasensitive electrochemical sensor for the detection and analysis of EGZ by electropolymerizing MI-PoPD onto the surface of PGE. Although EGZ itself is not electroactive, it was successfully detected through indirect molecular recognition. In this process, EGZ competes with the redox-active probe ([Fe(CN)₆]^3−/4−^) for binding sites within the MI-PoPD layer. The sensor was characterized using CV, EIS, and SEM coupled with EDX. The proposed sensor demonstrated excellent selectivity in the presence of co-formulated drugs, such as sitagliptin and metformin, and exhibited remarkable sensitivity, with a limit of detection (LOD) of 6.3 × 10⁻¹⁴ M. Furthermore, the method was assessed for its environmental impact using the GAPI and AGREE greenness assessment tools, which classified it as a green analytical approach. In conclusion, the proposed electrochemical sensor provides a versatile, highly sensitive, and environmentally friendly platform for the analysis of EGZ, showing significant potential for pharmaceutical applications.

## Supplementary Information

Below is the link to the electronic supplementary material.

Supplementary Material 1.

## Data Availability

The data sets used and analyzed are available from the corresponding author upon reasonable request.
