# Supplementary material for: Ultrasensitive, green molecularly-imprinted poly(o-phenylenediamine) sensor on pencil graphite for trace ertugliflozin quantification in plasma and tablets
Source: BMC Chem. 2025 Nov 29;19(1):317. doi: 10.1186/s13065-025-01681-1 (PMC12670783; doi:10.1186/s13065-025-01681-1)
Supplement: Supplementary file 1 — Supplementary Material 1. [file 13065_2025_1681_MOESM1_ESM.docx]

**Supplementary Information**

**for**

**Ultrasensitive, Green Molecularly-Imprinted Poly(o-phenylenediamine) Sensor on Pencil Graphite for Trace Ertugliflozin Quantification in Plasma and Tablets**

Menna Farrag ^1^, Sally S. El-Mosallamy ^2^, Bassam Shaaban Mohammed ^1,3^, Hytham Ahmed ^1,3*^

^1^ Pharmaceutical Analytical Chemistry Department, Faculty of Pharmacy, Menoufia University, Shebin Elkom, 32511, Egypt

^2^ Pharmaceutical Analytical Chemistry Department, Faculty of Pharmacy, Cairo University, Kasr El Aini, Cairo 11562, Egypt

^3^ Pharmaceutical Analytical Chemistry Department, Faculty of Pharmacy, Menoufia National University, 70^th^ Km Cairo-Alexandria Agricultural Road, Menoufia, Egypt

*Corresponding author: email: [hmaahmed@yahoo.co.uk](mailto:hmaahmed@yahoo.co.uk)

**
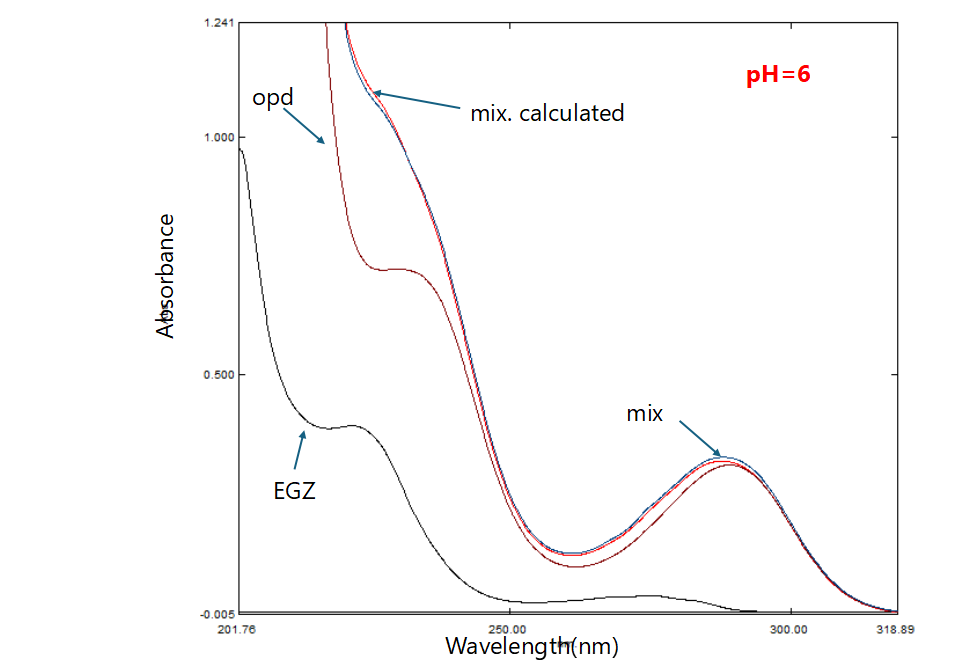
**

**
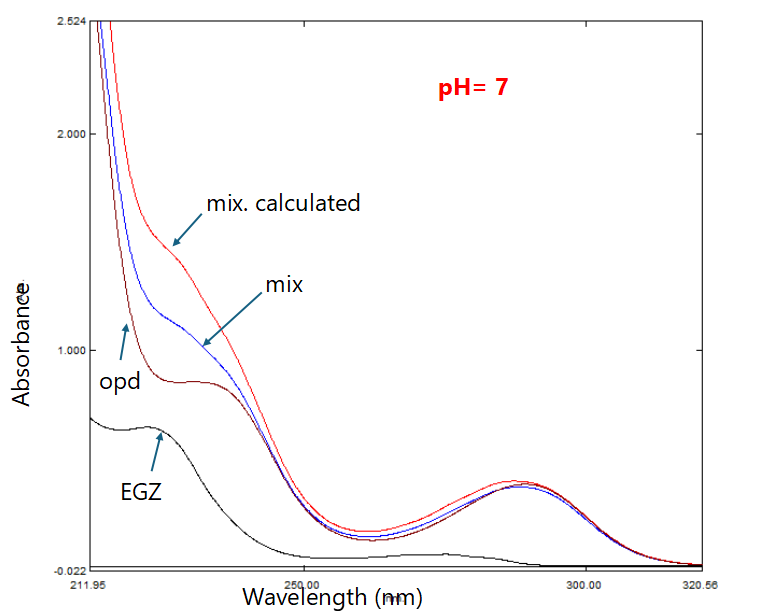
**

**
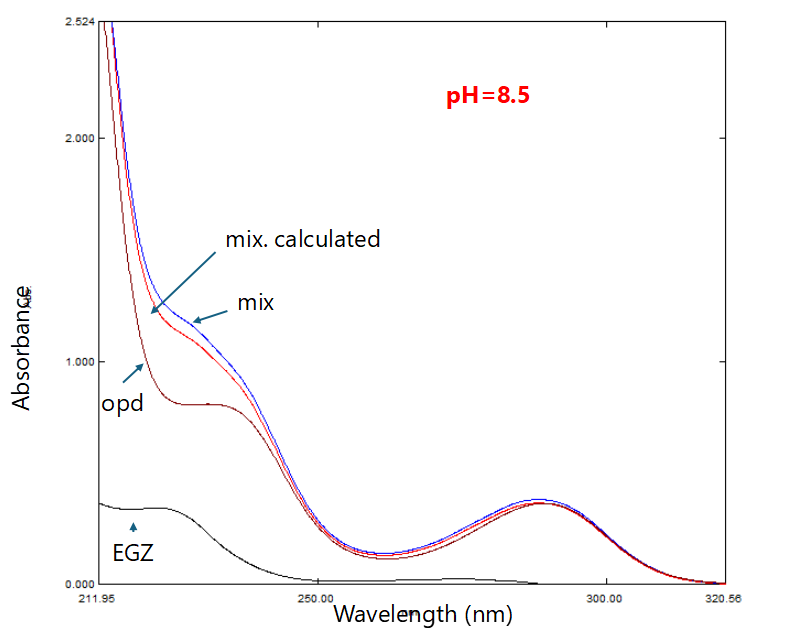
**

**Fig.S1. UV spectra of 1 × 10^-4^ M Ertugliflozin (EGZ) and the studied monomer o-phenylenediamine (o-PD) in different pH values of phosphate buffer (6 ,7 & 8.5) together with their 1:1 molar ratio pre-polymerization complexes.**

**
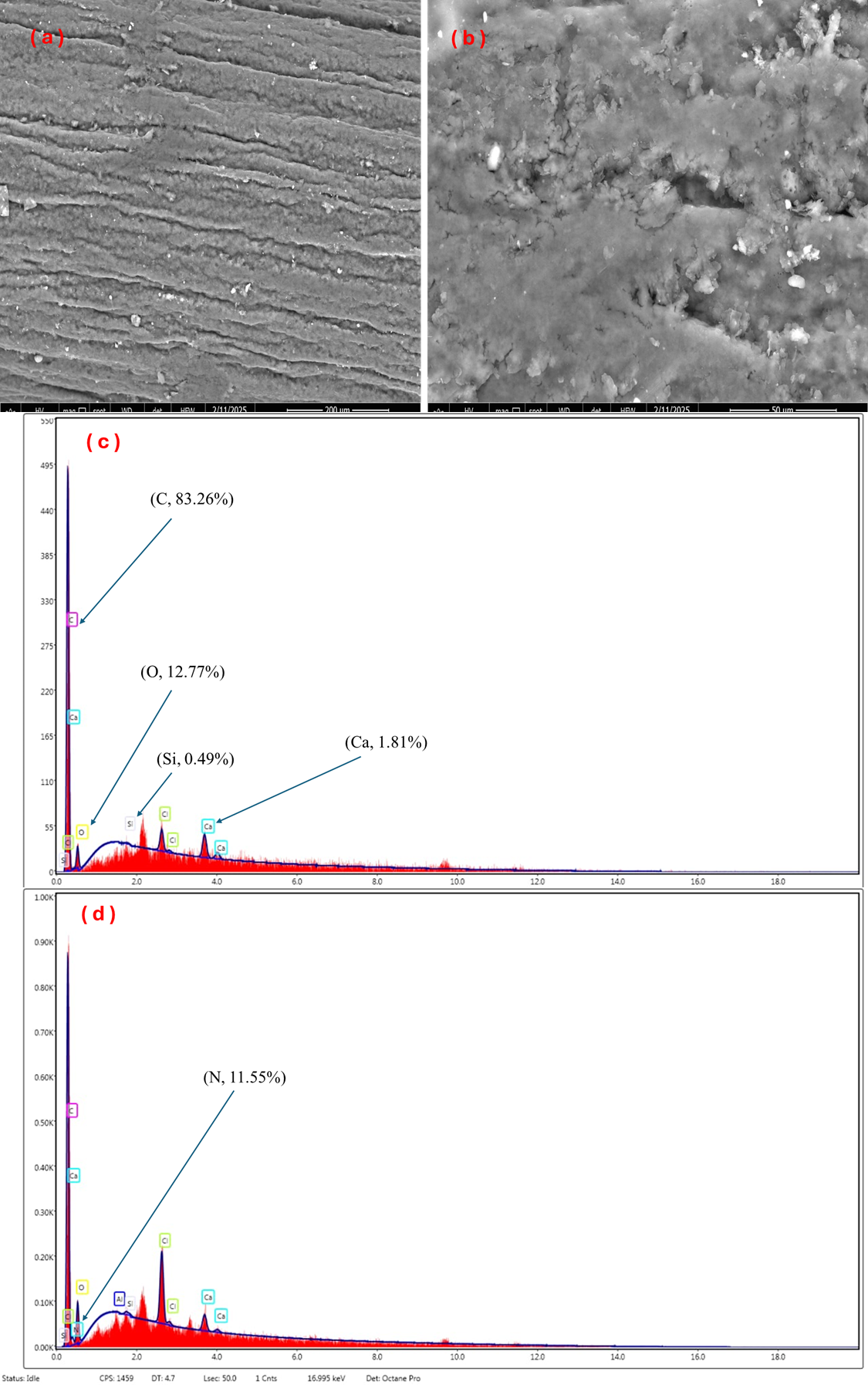
**

**Fig. S2. Scanning electron microscopy micrographs of (a) bare pencil graphite electrode and (b) poly(o-phenylenediamine)/pencil graphite electrode, and Energy Dispersive X-ray spectrum of (c) pencil graphite electrode and (d) poly(o-phenylenediamine)/pencil graphite electrode.**

**
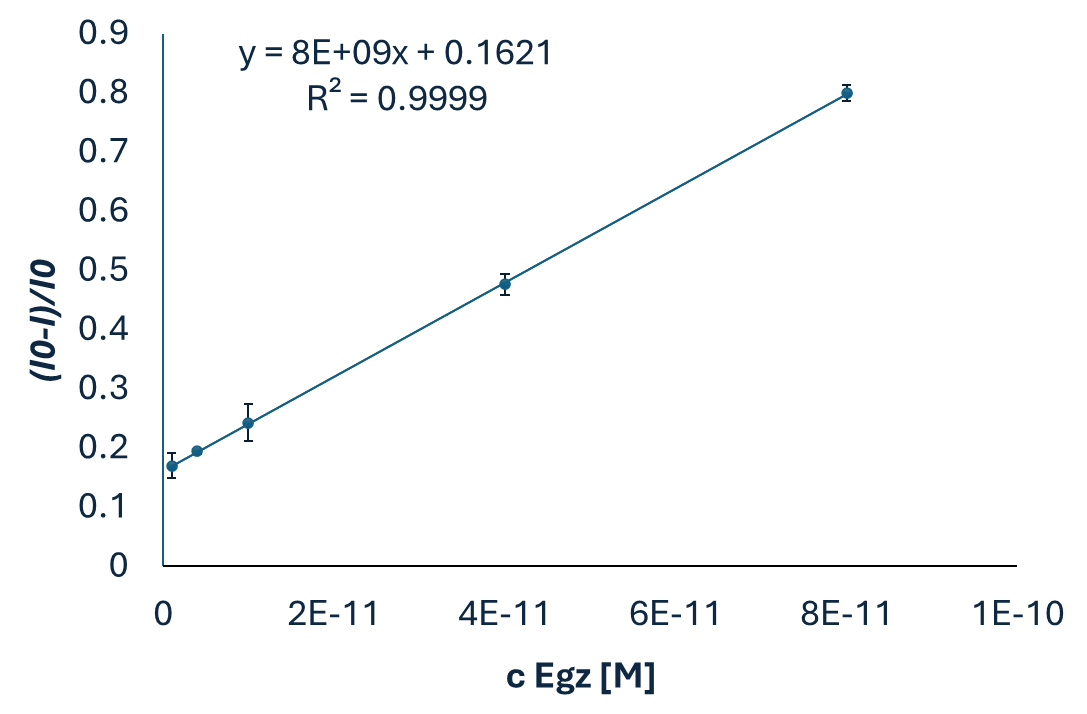
**

**Fig. S3. calibration curve of *(I^0^-I)/I^0^* versus the concentration of ertugliflozin (1 x 10^− 12^ – 1 x 10^− 10^M) using the optimized conditions.**

Table S4.

Comparison between the proposed MIP based electrochemical method and other reported methods for EGZ determination.

| **method** | **LOD** | **LOQ** | **Linear range** | **sample volume** | **matrix** | **selectivity scope** | **Run time** | **greenness scores (AGREE)** | **reference** |
| --- | --- | --- | --- | --- | --- | --- | --- | --- | --- |
| Spectrofluorimetry | 17.27 ng/mL | 52.32 ng/mL | 50 – 1000 ng/mL | Dosage form (tablets): Sample volume = 10 mL  Urine sample: Sample volume = 0.9 mL | Dosage form/Urine | -- | -- | 0.84 | [1] |
| NP-HPTLC | 7.17 ± 0.16 (ng/band) | 21.52 ± 0.48 (ng/band) | 50–600 ng/band | 10 µL (from stock solution) | Pharmaceutical tablets | Stability-indicating: separated ERZ from degradation products (acid, base, oxidative, thermal) | 8 cm plate development (~30 min chamber saturation + run) | 0.48 | [2] |
| RP-HPTLC | 0.94 ± 0.01 (ng/band) | 2.82 ± 0.03 (ng/band) | 25–1200 ng/band | 10 µL (from stock solution) | Pharmaceutical tablets | Stability-indicating: separated ERZ from degradation products (acid, base, oxidative, thermal) | 8 cm plate development (~30 min chamber saturation + run) | 0.89 |  |
| HPLC coupled with fluorescence detection | -- | 4 ng/mL | 4 – 2000 ng/mL | 100 µL rat plasma | Rat plasma | No endogenous interference at retention times | 20 min total per injection (isocratic elution, 1 mL/min) | -- | [3] |
| RP-HPLC | 0.21 µg/mL | 0.64 µg/mL | 0.64 – 20 µg/mL | Injection volume : 20 µL | Pharmaceutical tablets | Stability-indicating: both analytes resolved from degradation products; validated for precision, accuracy, robustness, and chemometric peak resolution | ~6 min | -- | [4] |
| RP-HPLC | -- | -- | 2.5 – 50 µg/mL | Injection volume :20 µL | Pharmaceutical tablets | Stability-indicating: ERZ peak resolved from degradation products under acid, base, oxidative, thermal, and photolytic stress | 6 min | -- | [5] |
| RP-HPLC | 12.71 µg/mL | 42.37 µg/mL | 37.5–112.5 µg/mL | Injection volume : 20 µL | Pharmaceutical tablets | Stability-indicating; degradants under acid, base, peroxide, hydrolytic, and UV stress resolved from sitagliptin | 6 min | -- | [6] |
| RP-HPLC | 0.13 µg/mL | 0.39 µg/mL | 3.75–22.5 µg/mL | Injection volume : 10 µL | Pharmaceutical tablets | Stability-indicating; clean separation from degradants under acid, base, oxidative, photolytic, thermal, and neutral stress | 5.0 min | -- | [7] |
| RP-HPLC | 1.2 µg/mL | 3.6 µg/mL | 6–14 µg/mL | Injection volume : 10 µL | Pharmaceutical tablets | Specific for ERZ in tablets (placebo showed no interference) | 8 minutes | -- | [8] |
| UPLC-MS/MS | -- | 1 ng/mL | 1 – 1000 ng/mL | 50 µL rat plasma | Rat plasma | No endogenous interference; validated for accuracy, precision, recovery, matrix effect, stability; applied to PK study | 3.0 min | -- | [9] |
| LC-MS/MS | -- | -- | 15 – 450 ng/mL | Injection volume : 5 µL | pharmaceutical dosage form | Specific, no interference from placebo | 6.0 min | -- | [10] |
| LC-MS/MS | -- | -- | 0.1–1.5 ng/mL | 200 µL plasma | Rat plasma | No endogenous interference | 5 min | -- | [11] |
| GC | ≈ 0.08% w/w vs L‑pidolic acid (≈ 0.02% w/w vs ertugliflozin) | 0.25% w/w vs L‑pidolic acid (≈ 0.06% w/w vs ertugliflozin) | 0.25–2.5% wt/wt versus L-pidolic acid or approximately 0.06–0.57% wt/wt of the  ertugliflozin drug substance sample. | Injection volume: 1 µL (split) | Ertugliflozin drug substance (cocrystal with L‑pidolic acid) | No interferences at D/L elution; clear baseline separation | ≈ 20–22 min to elute both enantiomers cleanly | -- | [12] |
| MIP electrochemical sensor | (3.6 x 10^-5^ ng/ml) | (1.08 x 10^-4^ ng/ml) | (5.66 x 10^-4^ – 5.66 x 10^-2^ ng/ml) | Dosage form (tablets): 25 ml  Plasma: 1 ml | Pharmaceutical dosage forms and blank human plasma | No interference from sitagliptin, metformin, dapagliflozin or other exciepients | -- | 0.59 | This work |

**Table S5**

**Green Analytical Procedure Index assessment of the proposed method’s greenness.**

| **Category** | **Proposed Method** |
| --- | --- |
| **Sample Collection (1)** | offline |
| **Sample Preservation (2)** | None |
| **Sample Transport (3)** | None |
| **Sample Storage (4)** | None |
| **Type of method (5)** | Simple Procedures |
| **Scale of extraction (6)** | Macro extraction |
| **Solvents/reagents used (7)** | green solvent |
| **Additional treatments (8)** | None |
| **Amount (9)** | 10-100 mL  (10-100 g) |
| **Health hazard (10)** | Potassium dihydrogen phosphate: NFPA health hazard score 0  Sodium Hydroxide: NFPA health hazard score 3  Acetic acid: NFPA health hazard score 3  Ethanol: NFPA health hazard score 2  Acetonitrile: NFPA health hazard score 2  o-phenylenediamine: NFPA health hazard score 3  Potassium ferrocyanide: NFPA health hazard score 1  Potassium ferricyanide: NFPA health hazard score 1 |
| **Safety hazard (11)** | Potassium dihydrogen phosphate: NFPA flammability score 0, instability score1  Sodium Hydroxide: NFPA flammability score 0, instability score 0  Acetic acid: NFPA flammability score 2, instability score 0  Ethanol: NFPA flammability score 3, instability score 0  Acetonitrile: NFPA flammability score 3, instability score 0  o-phenylenediamine: NFPA flammability score 1, instability score 0  Potassium ferrocyanide: NFPA flammability score 0, instability score 0  Potassium ferricyanide: NFPA flammability score 0, instability score 0 |
| **Energy (12)** | ≤0.1 kWh/ sample |
| **Occupational hazard (13)** | Hermetic sealing of analytical process (No vapors) |
| **Waste (14)** | >10mL |
| **Waste treatment (15)** | No Treatment |
| **Quantification** | Yes |

**Table S6**

**A statistical comparison between the proposed method and the reported method for pure powdered Ertugliflozin L-Pyroglutamic acid.**

| parameters | proposed method | reported method ^a^ |
| --- | --- | --- |
| Mean | 99.9 | 100.6 |
| SD | 1.4 | 3 |
| N | 5 | 5 |
| Variance | 1.96 | 9 |
| t-test | 0.473 (2.3060) ^b^ |  |
| F-value | 4.59 (6.39) ^b^ |  |

^a^ Ertugliflozin was determined using native fluorescence of the drug at λ_em_= 334 nm after excitation at λ_ex_ = 270 nm using water as a solvent [1].

^b^ Figures in parenthesis are the corresponding theoretical t and F values at (*p*=0.05)

**References**

1. Rasha M. Ahmed. Greenness assessment of spectrofluorometric method for quantification of ertugliflozin: application to dosage form and human urine. Rec Pharm Biomed Sci. 2021;5:135–44.

2. Alam P, Shakeel F, Alshehri S, Iqbal M, Foudah AI, Alqarni MH, et al. Comparing the greenness and validation metrics of traditional and eco-friendly stability-indicating HPTLC mMethods for ertugliflozin determination. ACS Omega. 2024;9:23001–12.

3. Han DG, Yun H, Yoon IS. A novel high-performance liquid chromatographic method combined with fluorescence detection for determination of ertugliflozin in rat plasma: assessment of pharmacokinetic drug interaction potential of ertugliflozin with mefenamic acid and ketoconazole. J Chrom B. 2019;1122–1123:49–57.

4. Murugesan A, Mukthinuthalapati Mathrusri A. Novel simplified, new analytical method for stress degradation study of ertugliflozin an oral anti-diabetic agent by RP-HPLC method. Acta Sci Pharm Sci. 2021;5:03–9.

5. Snigdha Rani Behera BKDSRM. Development and validation of a chemometric assisted analytical method for the simultaneous estimation of ertugliflozin and sitagliptin in pharmaceutical dosage forms by RP-HPLC. Adv Biores. 2023;14:156–65.

6. Babu DC, Chetty CM, Mastanamma SK. Novel stress indicating RP-HPLC method development and validation for the simultaneous estimation of ertugliflozin and sitagliptin in bulk and its formulation. Orient J Chem. 2018;34:2554–61.

7. Gurrala S, Shivaraj, Anumolu PD, Haripriya D, Subrahmanyam CVS. Chromatographic study of sitagliptin and ertugliflozin under quality-by-design paradigm. Braz J Pharm Sci. 2023;59:e21328.

8. Devika G.S, A Anjana, Isha MS, Enija E, Jeevitha T, Nevish P, et al. Determination of ertugliflozin in pharmaceutical formulations by RP-HPLC method. World J  Biol Pharm Health Sci. 2023;15:035–42.

9. Qiu X, Xie S, Ye L, Xu R ai. UPLC-MS/MS method for the quantification of ertugliflozin and sitagliptin in rat plasma. Anal Biochem. 2019;567:112–6.

10. Khoja SS, Patel LJ. Development and validation of new analytical LC-MS/MS method for the estimation of antidiabetic drugs ertugliflozin and sitagliptin in combined pharmaceutical dosage form. J Pharm Res Int. 2021;33:194–204.

11. Rao P, Rao A, Prasad S. Rapid quantitative estimation of metformin and ertugliflozin in rat plasma by liquid chromatography-tandam mass spectroscopy and its application to pharmacokinetic studies. Egypt Pharm J. 2021;20:1–7.

12. Nickerson B, Salisbury JJ, Harwood JW. Enantioselective analysis for l-pidolic acid in ertugliflozin drug substance and drug product by chiral gas chromatography with derivatization. J Pharm  Biomed Anal. 2018;159:212–6.
